# Research on metallic glasses at the atomic scale: a systematic review

**DOI:** 10.1007/s42452-022-05170-1

**Published:** 2022-09-30

**Authors:** Nicolás Amigo, Pablo Cortés, Felipe J. Valencia

**Affiliations:** 1 Facultad de Ingeniería, Arquitectura y Diseño, Universidad San Sebastián, Bellavista 7, Santiago, 8420524 Chile; 2 Independent Researcher, Tegualda 2000, 7770547 Ñuñoa, Chile; 3 Departamento de Computación e Industrias, Facultad de Ciencias de la Ingeniería, Universidad Católica del Maule, Talca, Chile; 4 Centro para el Desarrollo de la Nanociencia y la Nanotecnologa, CEDENNA, Avda. Ecuador 3493, Santiago, Chile

**Keywords:** Metallic glasses, Molecular dynamics, Bibliometric analysis, Systematic review

## Abstract

Metallic glasses (MGs) have been long investigated in material science to understand the origin of their remarkable properties. With the help of computational simulations, researchers have delved into structure-property relationships, leading to a large number of reports. To quantify the available literature, we employed systematic review and bibliometric analysis on studies related to MGs and classical molecular dynamics simulations from 2000 to 2021. It was found that the total number of articles has increased remarkably, with China and the USA producing more than half of the reports. However, high-impact articles were mainly conducted in the latter. Collaboration networks revealed that top contributor authors are strongly connected with other researchers, which emphasizes the relevance of scientific cooperation. In regard to the evolution of research topics, according to article keywords, plastic behavior has been a recurrent subject since the early 2000s. Nevertheless, the traditional approach of studying monolithic MGs at the short-range order evolved to complex composites with characterizations at the medium-range order, including topics such as nanoglasses, amorphous/crystalline nanolaminates, rejuvenation, among others. As a whole, these findings provide researchers with an overview of past and current trends of research areas, as well as some of the leading authors, productivity statistics, and collaboration networks.

## Introduction

Computational simulations in materials science has attracted wide attention during the last decades. The possibility to explore materials properties at different length scales, without incurring in high-cost experiments, has opened new horizons in the research community. Simulation methods, ranging from ab-initio to finite element modeling, have provided researchers with powerful techniques to inspect, design, and predict materials properties in the order of nanometers to milimeters [1, 2]. Classical molecular dynamics simulations, called molecular dynamics (MD) hereafter, is a simulation method based on Newtonian mechanics. Atomic interactions are modeled by means of interatomic potentials, usually parameterized to reproduce potential energy landscapes from experimental data or quantum calculations. Systems involving millions of atoms can be simulated at relatively low computational cost with high length-scale resolution, compared to quantum and continuum methods. Nevertheless, a disadvantage is that only small time-scales can be simulated and results strongly depend on the interatomic potential [3].

A wide variety of materials have been studied by means of MD in the literature. Some examples include biomaterials, ceramics, metals, polymers, and metallic glasses (MGs). MGs, often called amorphous metals, are novel materials successfully synthesized in 1960 [4, 5] that consist mainly of metallic elements with disordered atomic structure. Such combination leads to remarkable properties, including high strength, resistance to wear, and corrosion [6]. MD simulations have been performed to shed light on the atomistic mechanisms responsible for some of these properties. For example, studies on the glass forming ability include evolution of microstructure configurations of liquid metal systems under rapid cooling [7], atomic size effects on critical cooling rate for glass formation [8], and correlation between atomic structures and transport properties [9]. Another interesting topic is the mechanical behavior of MGs due to their lack of ductility and quasi-brittle behavior. However, previous reports indicated that large tensile ductility, with uniform elongation and extensive necking, can be observed in samples with dimensions in the order of 100 nm [10]. In order to elucidate the deformation behavior, researchers have investigated shear localization, shear transformation zones (STZs) nucleation, and shear band (SB) formation [11, 12]. Due to the lack of long-range order in amorphous materials, explanations to most of these phenomena rely on the short-range order (SRO) and medium-range order (MRO) atomic structure, usually characterized by means of Voronoi polyhedra and bond connectivity [13, 14].

There is a vast amount of literature available nowadays and it can be cumbersome to keep track of past and current investigations. Literature review is a traditional methodology for highlighting the most relevant discoveries, methodologies, and advances in a given research field. Several reviews on MGs can be found in the literature. For example, Cheng and Ma [15] explored 50 years of work devoted to unveil the structure-property relationship. Li et al. [16] summarized glass-producing techniques and discussed glass forming ability based on empirical rules and theory. Mechanical properties have also been surveyed. Egami et al. [17] compared simulations and experimental results of elastic, anelastic, and plastic behavior from an atomistic approach. All these reviews provide foundations, knowledge, and identification of open questions. Unfortunately, metrics related to the number of studies, authors, cites, among others, are usually out of the scope of such reports. Systematic literature review (SLR) is a method for identifying, evaluating, and synthesizing the work produced by researchers [18], whereas bibliometric analysis is a statistical evaluation of research documents [19]. Number of articles, collaboration networks, data inspection, and research methodologies are just a few examples of information gathered using SLR and bibliometric analysis in fields such as material selection processes [20], polymers manufacturing [21], biomaterials for implants [22], and smart glasses applications [23]. Here, through SLR and bibliometric analysis, we provide answers to the following questions on MGs studied by means of MD simulations: When did this research field grow? Where is the research performed? Who are the leading authors? What are the main topics under investigation? Due to the vast number of experimental and computational studies on MGs, the current work is limited to classical MD simulations as a first approach to conduct a SLR on this matter. This work is organized as follows. Section 2 presents the research framework together with the packages and tools employed during the analyzes. Section 3 shows the results, such as the evolution of this research field, the main journals, research trends, collaboration networks, among others. Section 4 discusses the results, their scope, and future works. Finally, Sect. 5 draws the conclusions.

## Methods

### Search strategy

**Fig. 1:**
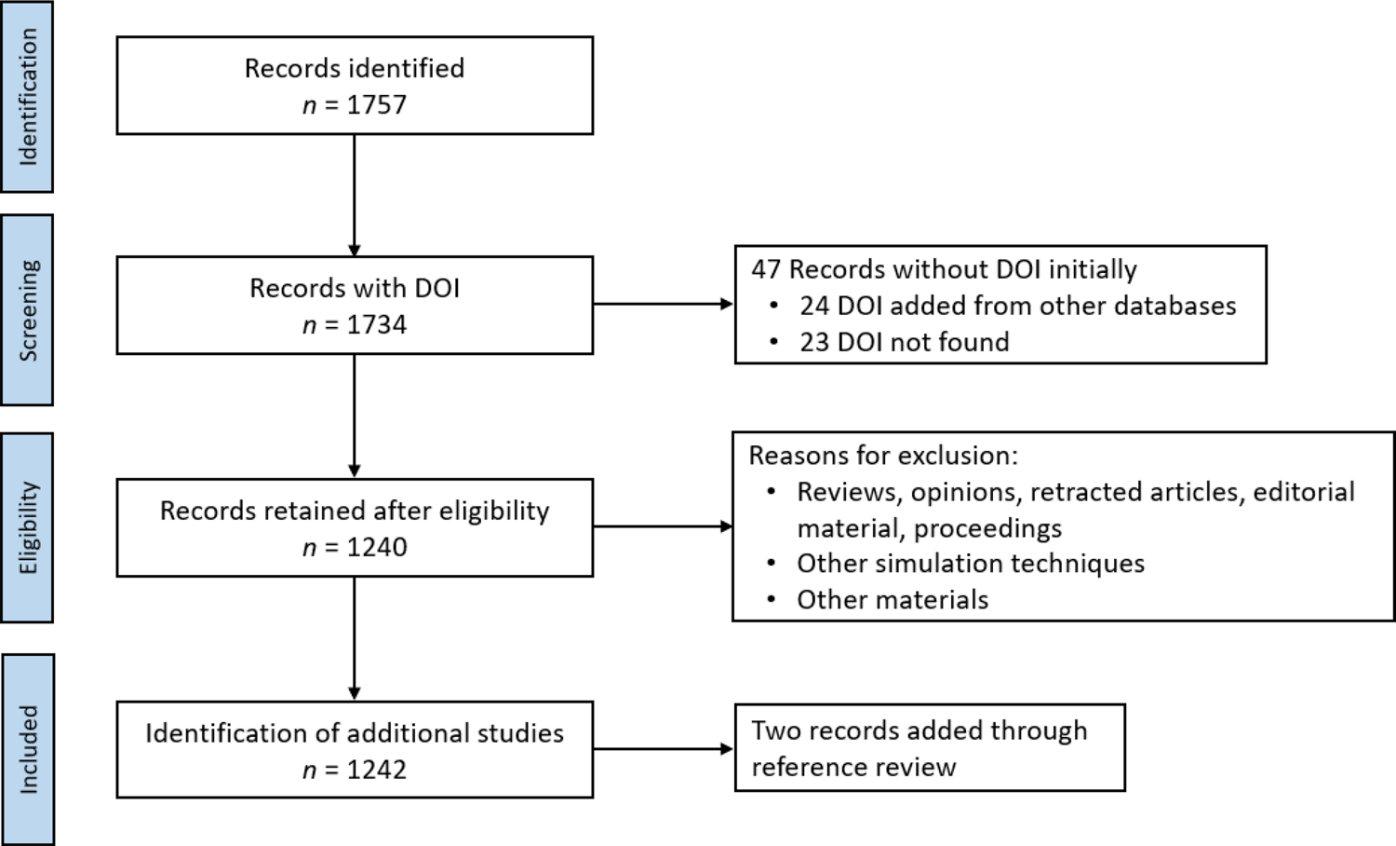
PRISMA flow diagram for the document selection process

This study was conducted and reported according to the Preferred Reporting Items for Systematic Reviews and Meta-Analyses Statement (PRISMA) [24] guidelines to ensure a structured and transparent review process as adopted elsewhere [25, 26]. The PRISMA flow diagram employed in this work is shown in Fig. 1.

Data acquisition was collected from a single data base as proposed by Merediz–Solà and Bariviera [27] to avoid systematic duplication. The Web of Science (WoS) was chosen for this purpose due to its prominence as a research tool [28]. The electronic search was conducted for English peer-reviewed studies published from January 1st, 2000 to October 26th, 2021. A boolean search strategy was adopted to relate two research areas: MGs and classical MD simulations. Since several synonyms have been coined for both areas, such as amorphous alloys, amorphous metals, atomistic simulations, among others, the following search strategy was employed:

$$\begin{aligned} {\text {Topic}}&= ({\text {metallic glass}}^{*} \,{\text {OR amorphous metal}}^{*}) \,{\text {AND}}\, ({\text {molecular dynamic}}^{*} {\text {OR atomistic simulation}}^{*}). \end{aligned}$$

A total of 1757 articles were obtained from the electronic search. It is worth to note that classical MD is usually referred as “molecular dynamics”. Nevertheless, this concept is much broader and it involves other techniques such as ab initio MD, molecular statics, among others. Exclusion of documents related to these techniques must be performed from direct inspection of the results.

### Data extraction

Several information parameters were extracted from the selected articles: (i) title, (ii) authors, (iii) journal, (iv) publication year, (v) corresponding author’s affiliation, (vi) research area, (vii) subject categories, (viii) keywords, (ix) WoS keyword plus, and (x) Digital Object Identifier (DOI). When not present, the DOI was manually retrieved from other online databases. Otherwise, the article was discarded. From DOI examination, a further 23 articles were discarded. A total of 1734 articles were retained after this procedure.

### Inclusion and exclusion criteria

The 1734 articles were screened based on a priori defined inclusion and exclusion criteria. Inclusion criteria for the systematic review were: (i) original article in the English language; and (ii) focus on metallic glasses and classical molecular dynamics simulations. Review articles, qualitative studies, letters, editorials, opinions, and conference abstracts were excluded. Early access articles were not considered due to the absence of publication year. Furthermore, articles focused on materials and simulation techniques different from MGs and classical MD simulations were also discarded.

Titles and abstracts were screened by one author (NA) for eligibility and a second author (FJV) checked pertinence of the results. If information in the title and abstract was insufficient to determine eligibility, full-text articles were inspected. Any disagreements or ambiguities were resolved through discussion. Finally, two additional articles were added through reference review, giving a total of 1242 documents for bibliometric analysis.

### Bibliometric analysis

Data analyzes were carried out to obtain performance indicators such as trends, number of articles, citations, top publishing journals, and country productivity. Topics coverage was inspected following article keywords. When not present, WoS KeyWords Plus were employed instead. Three periods of times were considered to assess the evolution of research topics: (i) 2000–2009, (ii) 2010–2019, and (iii) 2020–2021. Keywords related to MD simulations, MGs and their synonyms, such as atomistic simulation, amorphous metal, among others, were not considered to this aim. Collaboration networks among authors were constructed following network theory, where authors were represented by nodes and collaboration between two authors by edges. All analyzes were conducted using the R and Python programming languages, incluiding the Bibliometrix R package [29], Metaknowledge and, NetworkX Python packages [30, 31].

## Results

### Overview of search results

**Fig. 2:**
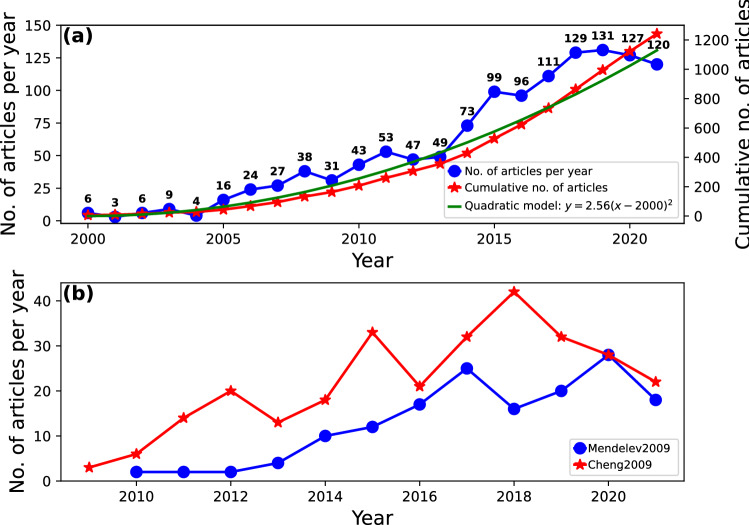
Number of articles published each year, accumulated number of articles, and analytical model for the growth of articles

Several studies on MGs and MD simulations have been conducted during the last decades. Figure 2a shows the number of articles published each year. As observed, just a few works were reported in the literature from 2000 to 2005, probably due to the limited computational power of that time together with the small research community devoted to MGs. Shortly after, the number of articles increased remarkably. Two explanations can be given to this phenomenon. The first one is the continuous enhancement of computational capability and speed [32] which have made accessible, for more researchers, to perform accurate and large scale molecular dynamics simulations. The second explanation can be found in the advances of MD simulations. In 2007 and 2009, Mendelev et al. [33, 34] published an interatomic potential to model CuZr MGs with high reliable structural characterizations at different atomic compositions. A few years later, Cheng et al. reported another potential to describe CuZrAl alloys [35]. Both contributions encouraged researchers to conduct a vast number of MD simulations on MGs. To quantify this matter, the number of articles referencing the Mendelev et al. [34] and Cheng et al. [35] works were retrieved, resulting in the curves shown in Fig. 2b. In both cases, the number of articles increased notoriously, reflecting the relevance of these models in the study of MGs. Unfortunately, the work of Mendelev et al. [33] is not available in the WoS database, making impossible to conduct an accurate analysis of references to this document. It is interesting to note that the total growth of articles in Fig. 2a, represented by the cumulative number of articles, closely resembles a quadratic function as shown by the green curve. Nevertheless, the number of publications dropped in 2020 and 2021. Similar drops also occurred in previous years (see for instance 2004, 2009, 2012, 2016), which suggests that this might be a temporary behavior until new research methodologies are developed. Another possibility is the COVID-19 impact as reported by Gao et al. [36]. Their study, based on surveys of principal investigators, revealed a decrease in initiating new research projects, suggesting that researchers are working on established topics. However, the real impact of COVID-19 pandemic is yet to be unveiled.

### Journals and research categories

**Fig. 3:**
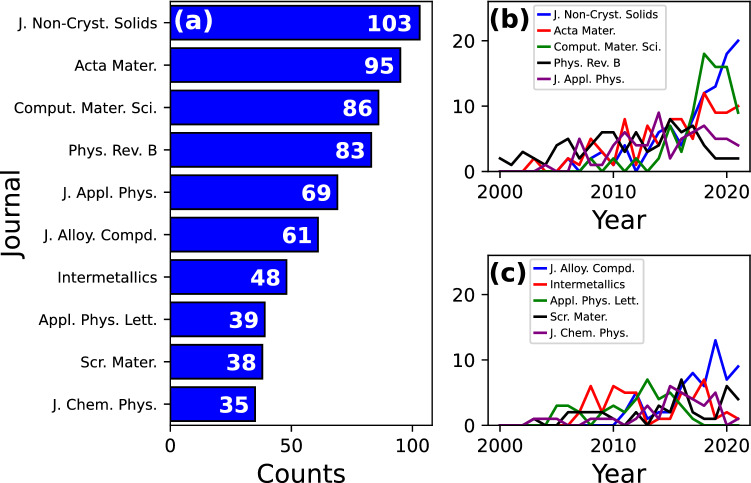
**a** Top ten journals with higher numbers of publications. **b**, **c** Evolution of the number of publications for each journal

Publications on MGs can be found in different high quality journals. Some of them are strongly committed to publish works related to this field, while others possess a wider scope. To shed light on this matter, Fig. 3a shows the top ten journals with most works related to MGs and MD simulations. The highest number of reports corresponds to the Journal of Non-Crystalline Solids ($n=103$), followed closely by Acta Materialia ($n=95$), Computational Materials Science ($n=86$), and Physical Review B ($n=83$). The first two of them are also focused on experimental studies and quite often their articles compare experimental results with computational simulations to better understand the observed phenomena. In contrast, Computational Materials Science is focused on computational methodologies and simulations, whereas a large number of articles in Physical Review B are based on condensed matter to gain insights into the underlying atomistic mechanisms in MGs. Figure 3b, c shows the evolution of the number of publications per year for each journal. The upward trend of Journal of Non-Crystalline Solids, Acta Materialia, Computational Materials Science, and Journal of Alloys and Compounds reflects the increasing interest in MGs among researchers. On the other hand, the number of studies in Physical Review B decayed from 2016 onwards, which can be explained from the launch of Physical Review Materials in 2017. This journal was conceived to cover several topics in materials science, such as synthesis, structure, modeling, among others [37]. Other well-reputed journals that publish on MGs and MD in a lesser degree include Journal of Applied Physics, Intermetallics, Applied Physics Letters, Scripta Materialia, and Journal of Chemical Physics.

**Fig. 4:**
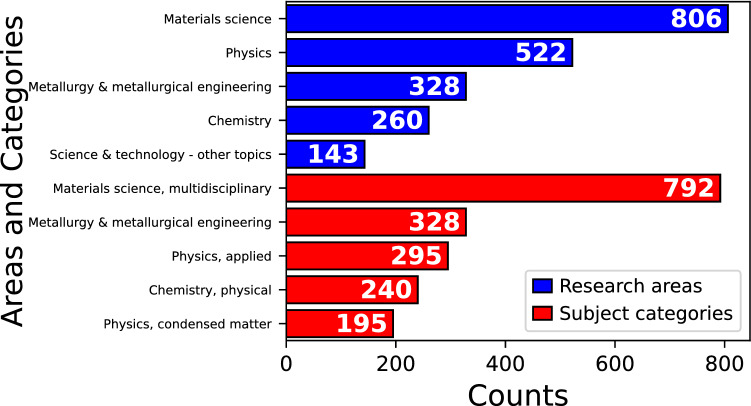
Research areas and subject categories of the top ten journals in publications on MGs and classical MD simulations

Research areas and subject categories are classifications that WoS assigns to all journals. The former delivers a general description of the topics covered by each journal, while the latter provides more details on the subjects. Both classifications were explored in our database obtaining the results shown in Fig. 4. In regard to research areas, most journals are classified as materials science ($n=806$), followed by physics ($n=522$) and then by metallurgy & metallurgical engineering ($n=328$). Such results are not surprising since MD simulations are frequently used to describe structural and mechanical properties of large-scale systems. Interestingly, subject categories reveal that physics is divided into two classifications: applied and condensed matter. The properties commented above usually fall into applied physics [38, 39]. However, other studies are focused on establishing theories and shedding light on fundamental mechanisms at the atomic scale which can be categorized as condensed matter. Some examples of these works can be found in Physical Review B [40, 41].

### Top publishing countries

**Fig. 5:**
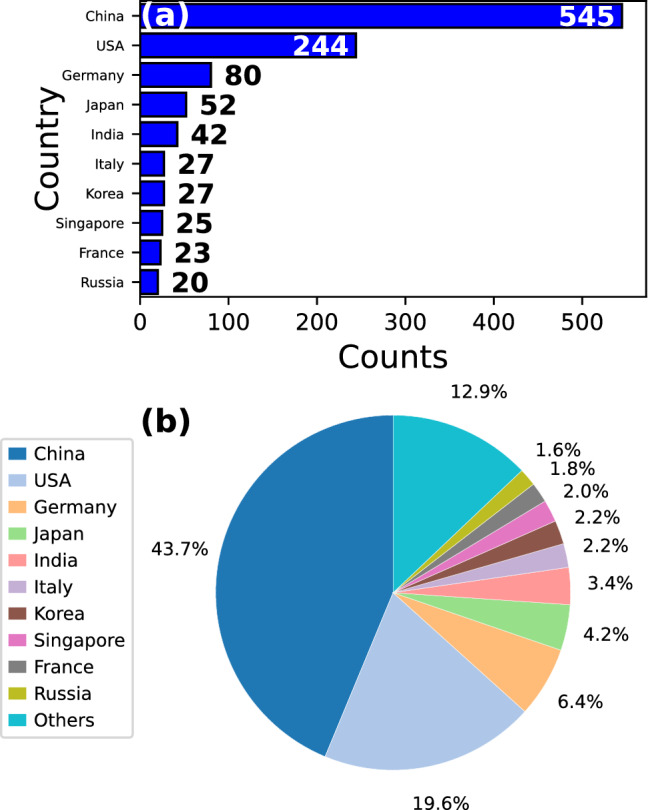
**a** Top ten countries in contributions on studies of MGs using MD simulations. **b** Percentage distribution per country. The “Others” wedge is composed by 27 countries with less than 20 articles

Investigations on MGs are carried out in many countries around the world. To elucidate their degree of contribution, the total number of articles per country was calculated by considering the corresponding author affiliation, resulting in the top ten list shown in Fig. 5a. A total of 545 articles correspond to China, representing almost half of the total as observed from Fig. 5b. The second country with most contributions is the USA with 244 articles, which represents a fifth of the total count. Germany, Japan, and India are the following nations with more than 30 articles. Productivity decays abruptly for other countries and 27 of them reported less than 20 articles (not shown in detail here), representing just 12.9% of the total number of works as observed in Fig. 5b. Possible factors for such disparity can be found in economic wealth, which has been identified to promote increased productivity as observed in high-income countries due to consolidation of research centers and institutions, strengthening of human capital, among others [42, 43]. Moreover, funding resources encourage mobility favoring international collaboration, scientific impact, and productivity [44]. Similar trends have also been reported in bibliometric analysis of nanotechnology [45]. The case of China has been discussed in the literature in detail. During the last years, China has invested in research and development more than the European Union. Its scientific institutions are now recognized worldwide and has more than 1.5 million researchers. Such factors have promoted the fast scientific growth of this nation and nowadays it has the largest productivity in research articles among all countries [46].

### Authors and collaborating networks

MD simulations on MGs is an increasing research field that has attracted the attention of many authors. Remarkable studies can be found in the literature. Table 1 shows the ten most cited articles during the last two decades. One of the earliest research fields were related to structural disorder of amorphous solids. As reflected from the works of Truskett et al. [47] and Sheng et al. [48], atomic packing of simple hard-sphere systems and binary alloys was thoroughly inspected to quantify both the SRO and the MRO. A common topic also covered is the mechanical behavior of MGs. Brittle-to-ductile transition has been explored by determining the critical stress for SB nucleation [49], enhancement of ductility in crystalline-amorphous nanolaminates [50], and identification of the local environment of shear localization [51–53]. Development of accurate interatomic potentials are essential to conduct proper MD simulations, as reflected from the works of Mendelev et al. [33, 34]. To this aim, the authors developed models for CuZr alloys. Publication and availability of such potentials provided researchers with state-of-the-art techniques to explore CuZr MGs, which led to novel studies as will be discussed in the following sections. Formation of monoatomic MGs is also a topic of interest as demonstrated by the work of Zhong et al. [54]. It was long believed that monoatomic MGs were experimentally unfeasible to synthesize due to the high cooling rate required for vitrification. In a remarkable effort, the authors showed that liquid tantalum and vanadium were successfully vitrified by employing cooling rates in the order of 10$^{14}$ K/s and the dynamic process was unveiled by means of MD simulations. All of these studies were mainly conducted in the USA, which indicates that despite its second place in the number of publications (see Fig. 5a), the USA contributes with higher impact articles in the field.

**Table 1:** Articles with most cites from 2000 to 2021

References	Year	Journal	Times cited	Country
Sheng et al. [48]	2006	Nature	1405	USA
Shimizu, et al. [49]	2007	Mater. Trans.	599	USA, Japan
Wang et al. [50]	2007	Proc. Natl. Acad. Sci. USA	337	USA
Cheng et al. [52]	2008	Acta Mater.	321	USA
Cao et al. [51]	2009	Acta Mater.	291	USA
Mendelev [34]	2009	Philos. Mag. Lett.	273	USA, Russia
Zhong et al. [54]	2014	Nature	252	USA, China
Cheng et al. [53]	2009	Acta Mater.	245	USA
Truskett [47]	2000	Phys. Rev. E	239	USA
Mendelev [33]	2007	J. Appl. Phys.	234	USA

**Fig. 6:**
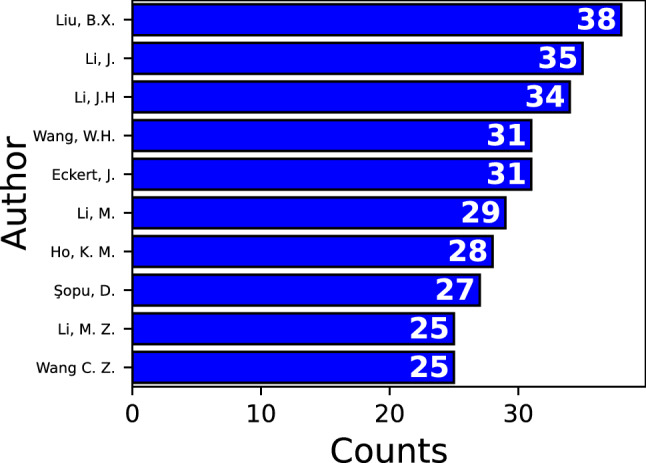
Top ten authors on MGs and classical MD simulations

Some authors have been involved to a greater extent than others when studying MGs. A list of the top ten authors with most articles is presented in Fig. 6. Their efforts have been directed to explore different properties of MGs. For example, glass forming ability together with interatomic potential implementation have been explored by Liu and Li [55, 56]. Mechanical properties have been studied by Şopu, D. and Eckert, J., reporting nanoglasses and nanocomposites with enhanced ductility [57, 58]. Shear banding theory, including critical size for nucleation, propagation, localization, and atomic scale characterization have been developed by Li [11, 59] and Li [60, 61]. Finally, several works on atomic packing, structural heterogeneity, and MRO have been reported by Li et al. [62–64]. All of these authors have also contributed in other topics on MGs using MD simulations. However, it is out of the scope of the present work to give an exhaustive list, and thus, only some of their contributions are mentioned here.

**Fig. 7:**
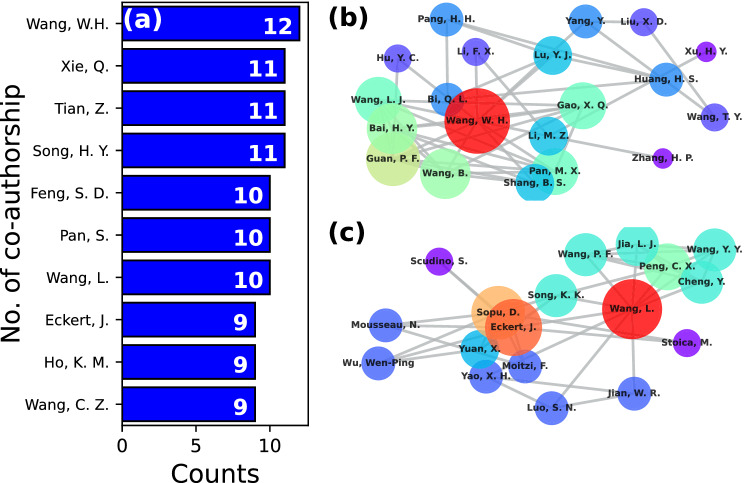
**a** Top ten authors with higher number of co-authors in the time period from 2017 to 2021. **b**, **c** The two main components of the co-authors network. Large node size and red color correspond to high collaboration, while small size and purple to low collaboration

Previous studies have reported that higher impact research can be achieved by means of larger collaboration networks [65, 66]. Co-authors network was constructed from the literature survey performed in this work. Due to the large number of articles, only collaborations from 2017 to 2021 were considered. Analysis of this period of time represents the current co-authorship in the field. A large number of collaborations were obtained from the records and many of them were composed by a few authors. In order to simplify the discussion and to give a general overview, collaborations with less than two articles were excluded. In the collaboration network, the number of co-authors for each author was obtained from the number of edges connected to each node, resulting in the top ten authors with most collaborators shown in Fig. 7a. Wang, W. H. stands out as the author with most co-authors ($n = 12$) during the last five years, followed closely by Xie, Q., Tian, Z., and Song, H. Y. ($n=11$). Some of them, such as Wang, W. H., Eckert, J., and Wang, C. Z., are also top ten contributing authors as commented previously from Fig. 6, which highlights the relevance of scientific cooperation. More details of collaboration can be obtained from direct inspection of the networks. Since the full network is composed of many (disconnected) subnetworks, only the two largest components are shown here. Figure 7b presents the largest subnetwork with a total of 20 authors, where a large node size and red color correspond to authors with a high number of co-authors. Here, Wang, W.H. serves as a hub that connects other researchers, explaining his large number of publications. Some works reported by this group are in the field of relaxation dynamics [67] and glass-forming ability [68]. In the second largest component, shown in Fig. 7c, Wang, L. is identified as a hub. Interestingly, this subnetwork also finds two other relevant hubs in Eckert, J. and Sopu, D., indicating strong collaboration among these authors. Some of their works include creep behavior [69] and structural heterogeneity [70]. Therefore, leading authors are strongly supported by other scientists, which is vital to conduct high impact research.

### Research trends

**Fig. 8:**
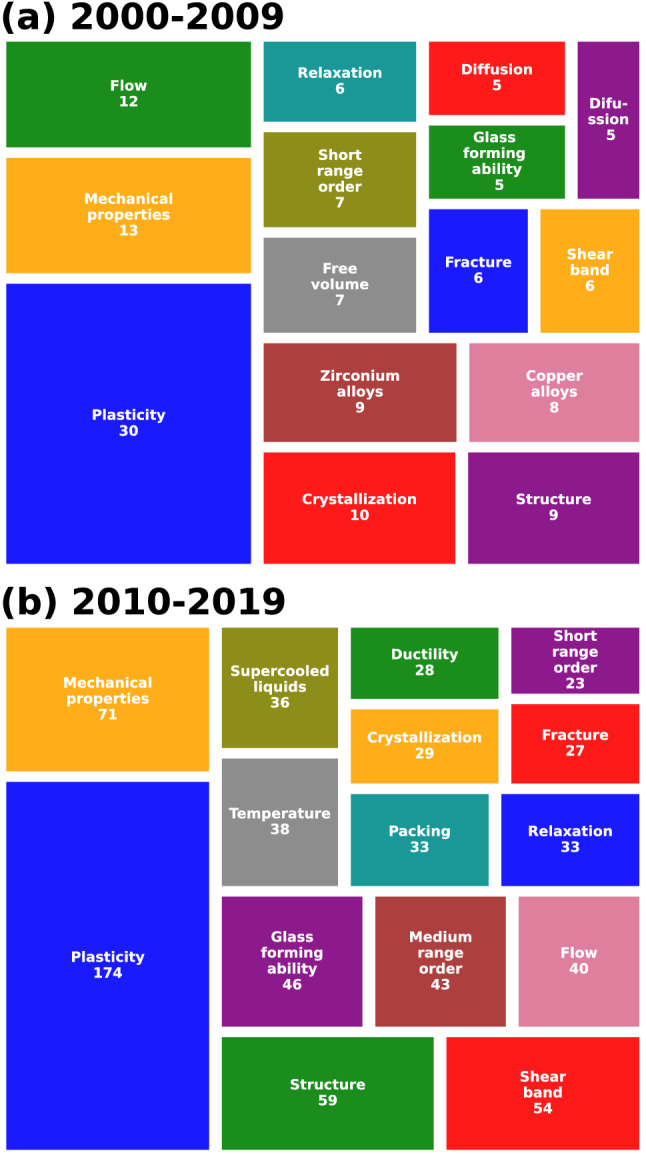
Most common keywords **a** from 2000 to 2009 and **b** from 2010 to 2019

Research topics on MGs by means of MD simulations have varied across the years. Early studies were frequently based on the atomic packing of amorphous metals, while the recent literature comprise a wide variety of approaches. Articles keywords were explored to summarize the main topics addressed in the last two decades. Figure 8a, b present the treemaps with the 15 most frequent keywords corresponding to articles running from 2000 to 2009 and from 2010 to 2019, respectively. The number in each box corresponds to the number of counts. Since the retrieved keywords were not abbreviated, their full form are shown in the treemaps. In the following, brief descriptions of the surveyed literature are given according to the topics shown in Fig. 8. No in-depth details are provided since the current work is focused on quantitative analysis. The reader is referred to specialized literature reviews for more in-depth discussions [15–17, 71].

Large interest in mechanical behavior has been present from 2000 to 2019, as represented by keywords such as “Plasticity”, “Mechanical properties”, “Flow”, “Ductility”, and “Fracture”. Back to the 2000s, a common practice was to adopt simple Lennard–Jones based models to simulate monolithic MGs, probably due to the lack of realistic potentials to describe amorphous solids. Some examples include monatomic Lennard–Jones glasses [72], polydisperse, two-dimensional, noncrystalline models [73], and 80%–20% binary mixtures of Lennard–Jones particles [74]. Despite this drawback, researchers performed successful simulations of crack tip deformation [73], tension-compression tests to explore asymmetries in yield stress [75], stress–strain dependence on physical aging, shear rate and temperature [74], shear localization and SB characterization [11], and critical conditions for SB maturation [60]. In 2007 and 2009, Mendelev et al. [33, 34] and Cheng et al. [35] constructed interatomic potentials based on the embedded atom method (EAM) to model CuZr and CuZrAl MGs, which explains the “Zirconium alloys” and “Copper alloys” keywords displayed in Fig. 8a. All these reports paved the way for more complex studies during the following decade. Some examples include shock wave loading [76, 77], crystalline-amorphous nanocomposites [78], shape memory alloy reinforced composites [57, 79], and structural heterogeneities for tuning the plastic behavior [80]. An estimation of the number of times that each alloy has been explored in the literature can be obtained from direct inspection of the abstracts. It was found that CuZr MGs were the most studied ($n=165$), followed by CuZrAl MGs ($n=28$), NiZr MGs ($n=20$), and monoatomic MG ($n=3$). However, the number of times for each case is probably underestimated, since quite often the abstract does not explicitly indicate the alloy under consideration. A more in-depth analysis of the documents can amend these values, as well as help to identify other alloys under study.

Another topic of interest is the vitrification process to obtain amorphous solids as depicted by the “Glass forming ability”, “Crystallization”, “Supercooled liquids”, “Diffusion”, and “Relaxation” keywords. Depending on stoichiometry, metal alloys exhibit different degrees of glass forming ability. Unfortunately, its prediction is still a task to be fulfilled. During both decades, ideal and realistic systems were explored, including studies on structural relaxation and packing density [81], competing order between liquid and crystal phases [82], and self-diffusion coefficient and relaxation time [83].

Materials properties are ultimately determined by the atomic structure. Hence, most studies have been focused on correlating the disordered structure of MGs with their macroscopic properties as reflected from the “Short range order” and “Free volume” keywords in Fig. 8a. The former describes the local environment of atoms and has been employed to establish the structural backbone of MGs [52, 84]. The latter is considered an important concept in plasticity, since free volume generation and localization leads to SB formation [11, 85]. From 2010 to 2019, more attention was paid to the “Medium range order” as shown in Fig. 8b. Connectivity of atomic clusters corresponds to MRO analysis. By inspecting such structures, researchers elucidated the second split observed in the pair distribution function [86] and determined enhanced resistance to deformation due to interpenetrating networks of full icosahedra [87].

**Fig. 9:**
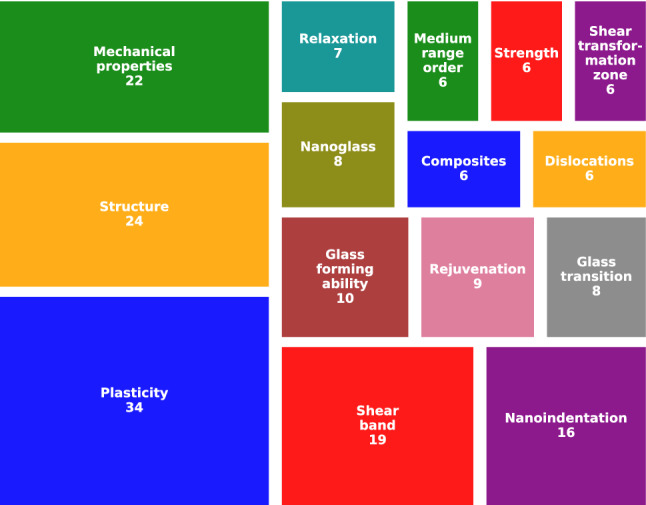
Most common keywords from 2020 to October 26th, 2021, the date of the literature survey

New research topics have emerged in the literature from 2020 to 2021 as shown in Fig. 9, where mechanical behavior studies are still dominant. Keywords such as “Rejuvenation”, “Nanoindentation”, “Nanoglass”, and “Dislocations” are now present. Rejuvenation refers to the procedure devoted to drive MGs to higher energy levels. Different methods at the atomic scale have been reported, including elastostatic compression [88], low-energy ion irradiation [89], thermal cycling between ambient and cryogenic temperatures [90], and pressure-promoted treatments [91]. Indentation is a mechanical test to measure material hardness, and at the atomic scale it has been performed on amorphous/amorphous [92] and amorphous/crystalline nanolaminates [93], whose plastic deformation leads to dislocation nucleation and enhanced ductility. Since simulation of composites involves a large number of atoms, reports on this topic have become more frequent probably due to the increased computational power. Nowadays it is quite common to find novel studies of large-scale systems, such as nanocutting of both amorphous [94] and amorphous/crystalline composites [95], tensile tests of shape memory metallic glass composites with brick and mortar designs [96], and shear localization in nanoglasses with gradient design [97].

## Discussion

Research studies on MGs by means of MD simulations have been evolving remarkable during the last two decades. In the early 2000s, less than ten articles were published each year. However, computer advances, development of novel techniques, growth of the scientific community, among others, has led to the publication of more than a hundred articles. It is difficult to deliver an accurate quantification of the total number of studies, since other document types such as proceedings and reviews were not included here, and some of them do not have a DOI. Scientific journals have also evolved through these years. In the beginning, studies on both MGs and MD simulations were published almost evenly in different journals, but from 2010 onwards, some of them acquired more relevance in this field, such as Acta Materialia, Journal of Non-Crystalline Solids, Journal of Alloys and Compounds, and Computational Materials Science. Many studies available in the aforementioned journals are focused on MGs using solely experimental approaches. Since such studies were not considered in this work, the real contribution of each journal to the field may be underestimated. An interesting approach would be to compare the total number of experiment-based studies with those of simulation-based as a method to estimate the types of productivity of authors and countries.

Contribution of each country was calculated by considering the corresponding author affiliation, resulting in that China and the USA are the most prominent contributors. This result could change by including also the country of each co-author. Furthermore, international cooperation can be established from direct inspection of affiliations and, with the help of network analysis, prominent research institutions can be identified. Similar methodologies on this matter have been applied in other research fields, for example, in solar energy [98], COVID-19 [99], and even in more specific areas such as density functional theory [100]. Unfortunately, construction of these networks requires more in-depth calculations being out of the scope of the current work.

Collaboration networks delivered a qualitative overview of the two largest components of co-authorship. To shed light on other collaborations, more components must be explored. Since the size of the network is explicitly related to the number of authors and implicitly related to the number of articles, such procedure cannot be conducted by direct visual inspection. To overcome this issue, networks metrics should be calculated. For example, betweenness centrality can unveil the degree of influence of each author over the network, and macroscopic metrics, such as density and transitivity, can be employed to determine the connectivity degree of both networks and subnetworks. Although our analysis was restricted to the number of co-authors, other networks can also be explored. As pointed out in other works [101, 102], keywords, author co-citation, journal co-citation, among others, can provide insights into relevant research facilities, influential literature, cooperation, and the latest frontier in the field.

## Conclusions

Metallic glasses and classical molecular dynamics simulations is a long-standing research area with a vast amount of studies that range from glass formation to mechanical properties of complex nanolaminates. This systematic review provided quantitative analyzes of the literature published from 2000 to 2021, giving insights into the number of articles, journals, research areas, collaboration networks, among others. Our results revealed that this research field has gained increased attention over the years. The number of articles has growth remarkably and several specialized journals are publishing a large number of works. Most of these investigations are conducted in a few countries, indicating that cutting-edge research is highly localized. In addition, leading authors present a large number of co-authors as reflected from the collaboration networks. Regarding the evolution of research topics, early studies were strongly focused on simple models of amorphous studies to study plastic behavior and glass forming ability with emphasis in the short-range order. However, with the advances of interatomic potentials and better understanding of the structural properties, more complex studies emerged, with focus on amorphous/crystalline composites, medium-range order, rejuvenation, among others.

Overall, the number of possible studies that can be derived from systematic reviews and bibliometric analyzes is vast. Number of articles, leading authors, research topics, and collaboration networks are just a few examples. All together can unveil and dissect the current trends in materials science, benefiting scholars, young researchers, and senior researchers to establish new research projects.

## Data Availability

The datasets used and/or analyzed during the current study are available from the corresponding author on reasonable request.

## References

[1] Steinhauser MO, Hiermaier S (2009). A review of computational methods in materials science: examples from shock-wave and polymer physics. Int J Mol Sci.

[2] Elliott JA (2011). Novel approaches to multiscale modelling in materials science. Int Mater Rev.

[3] Rapaport DC (2004). The art of molecular dynamics simulation.

[4] Duwez P, Willens RH, Klement W (1960). Continuous series of metastable solid solutions in silver-copper alloys. J Appl Phys.

[5] Klement W, Willens RH, Duwez P (1960). Non-crystalline structure in solidified gold-silicon alloys. Nature.

[6] Johnson WL (1996). Bulk metallic glasses—a new engineering material. Curr Opin Solid State Mater Sci.

[7] Rangsu L, Jiyong L, Kejun D, Caixing Z, Hairong L (2002). Formation and evolution properties of clusters in a large liquid metal system during rapid cooling processes. Mater Sci Eng B.

[8] Jalali P, Li M (2005). Atomic size effect on critical cooling rate and glass formation. Phys Rev B.

[9] Zhang Y, Mattern N, Eckert J (2012) Understanding the relationship between atomic structures and transport properties in (cu$$_{0.5}$$zr$$_{0.5}$$)$$_{100-x}$$al$$_x$$ ($$<$$10) glass forming liquids: Molecular dynamics simulations. J Alloys Compd 514:141–149. 10.1016/j.jallcom.2011.11.034

[10] Guo H, Yan PF, Wang YB, Tan J, Zhang ZF, Sui ML, Ma E (2007). Tensile ductility and necking of metallic glass. Nat Mater.

[11] Li QK, Li M (2006). Atomic scale characterization of shear bands in an amorphous metal. Appl Phys Lett.

[12] Zink M, Samwer K, Johnson WL, Mayr SG (2006). Plastic deformation of metallic glasses: Size of shear transformation zones from molecular dynamics simulations. Phys Rev B.

[13] Wakeda M, Shibutani Y (2010). Icosahedral clustering with medium-range order and local elastic properties of amorphous metals. Acta Mater.

[14] Lee M, Lee CM, Lee KR, Ma E, Lee JC (2011). Networked interpenetrating connections of icosahedra: Effects on shear transformations in metallic glass. Acta Mater.

[15] Cheng Y, Ma E (2011). Atomic-level structure and structure-property relationship in metallic glasses. Prog Mater Sci.

[16] Li J, Dai Y, Cui Y, Liu B (2011). Atomistic theory for predicting the binary metallic glass formation. Mater Sci Eng R Rep.

[17] Egami T, Iwashita T, Dmowski W (2013). Mechanical properties of metallic glasses. Metals.

[18] Fink A (2019). Conducting research literature reviews: from the Internet to paper.

[19] Broadus RN (1987). Toward a definition of “bibliometrics”. Scientometrics.

[20] Rahim AA, Musa SN, Ramesh S, Lim MK (2020). A systematic review on material selection methods. Proc Inst Mech Eng Part L J Mater Des Appl.

[21] Zheng Y, Zhang W, Baca Lopez DM, Ahmad R (2021). Scientometric analysis and systematic review of multi-material additive manufacturing of polymers. Polymers.

[22] Kohli N, Stoddart JC, van Arkel RJ (2021). The limit of tolerable micromotion for implant osseointegration: a systematic review. Sci Rep.

[23] Kim D, Choi Y (2021). Applications of smart glasses in applied sciences: A systematic review. Appl Sci.

[24] Moher D, Liberati A, Tetzlaff J, Altman DG (2009). Preferred reporting items for systematic reviews and meta-analyses: the prisma statement. BMJ.

[25] Diehl K, Thiel A, Zipfel S, Mayer J, Litaker DG, Schneider S (2012). How healthy is the behavior of young athletes? A systematic literature review and meta-analyses. J Sports Sci Med.

[26] Liao Y, Deschamps F, de Freitas Rocha Loures E, Ramos LFP (2017) Past, present and future of industry 4.0—a systematic literature review and research agenda proposal. Int J Prod Res 55(12):3609–3629. 10.1080/00207543.2017.1308576

[27] Merediz-Solá I, Bariviera AF (2019). A bibliometric analysis of bitcoin scientific production. Res Int Bus Financ.

[28] Li K, Rollins J, Yan E (2018). Web of science use in published research and review papers 1997–2017: a selective, dynamic, cross-domain, content-based analysis. Scientometrics.

[29] Aria M, Cuccurullo C (2017). bibliometrix: An r-tool for comprehensive science mapping analysis. J Informet.

[30] McLevey J, McIlroy-Young R (2017). Introducing metaknowledge: Software for computational research in information science, network analysis, and science of science. J Informet.

[31] Hagberg AA, Schult DA, Swart PJ (2008) Exploring network structure, dynamics, and function using networkx. In: Varoquaux G, Vaught T, Millman J (eds) Proceedings of the 7th Python in science conference, Pasadena, CA USA, pp 11 – 15

[32] Schaller R (1997). Moore’s law: past, present and future. IEEE Spectr.

[33] Mendelev MI, Sordelet DJ, Kramer MJ (2007). Using atomistic computer simulations to analyze X-ray diffraction data from metallic glasses. J Appl Phys.

[34] Mendelev M, Kramer M, Ott R, Sordelet D, Yagodin D, Popel P (2009). Development of suitable interatomic potentials for simulation of liquid and amorphous Cu–Zr alloys. Philos Mag.

[35] Cheng YQ, Ma E, Sheng HW (2009). Atomic level structure in multicomponent bulk metallic glass. Phys Rev Lett.

[36] Gao J, Yin Y, Myers KR, Lakhani KR, Wang D (2021). Potentially long-lasting effects of the pandemic on scientists. Nat Commun.

[37] Physical review materials. https://journals.aps.org/prmaterials/about

[38] Li QK, Li M (2007). Assessing the critical sizes for shear band formation in metallic glasses from molecular dynamics simulation. Appl Phys Lett.

[39] Adibi S, Sha ZD, Branicio PS, Joshi SP, Liu ZS, Zhang YW (2013). A transition from localized shear banding to homogeneous superplastic flow in nanoglass. Appl Phys Lett.

[40] Zaccone A, Scossa-Romano E (2011). Approximate analytical description of the nonaffine response of amorphous solids. Phys Rev B.

[41] Milkus R, Zaccone A (2016). Local inversion-symmetry breaking controls the boson peak in glasses and crystals. Phys Rev B.

[42] Männasoo K, Hein H, Ruubel R (2018). The contributions of human capital, R &D spending and convergence to total factor productivity growth. Reg Stud.

[43] Jaffe K, ter Horst E, Gunn LH, Zambrano JD, Molina G (2020). A network analysis of research productivity by country, discipline, and wealth. PLoS ONE.

[44] Netz N, Hampel S, Aman V (2020). What effects does international mobility have on scientists’ careers? A systematic review. Res Eval.

[45] Pisarenko Z V, Ivanov LA, Wang Q (2020) Nanotechnology in construction: state of the art and future trends. Nanotechnol Constr A Sci Int J 12(4):223–231. 10.15828/2075-8545-2020-12-4-223-231

[46] Van Noorden R (2016). China by the numbers. Nature.

[47] Truskett TM, Torquato S, Debenedetti PG (2000). Towards a quantification of disorder in materials: distinguishing equilibrium and glassy sphere packings. Phys Rev E.

[48] Sheng HW, Luo WK, Alamgir FM, Bai JM, Ma E (2006). Atomic packing and short-to-medium-range order in metallic glasses. Nature.

[49] Shimizu F, Ogata S, Li J (2007). Theory of shear banding in metallic glasses and molecular dynamics calculations. Mater Trans.

[50] Wang Y, Li J, Hamza AV, Barbee TW (2007) Ductile crystalline-amorphous nanolaminates. Proc Natl Acad Sci 104(27):11,155–11,160. 10.1073/pnas.0702344104 10.1073/pnas.0702344104 PMC1899185 17592136

[51] Cao A, Cheng Y, Ma E (2009). Structural processes that initiate shear localization in metallic glass. Acta Mater.

[52] Cheng Y, Cao A, Sheng H, Ma E (2008). Local order influences initiation of plastic flow in metallic glass: effects of alloy composition and sample cooling history. Acta Mater.

[53] Cheng Y, Cao A, Ma E (2009). Correlation between the elastic modulus and the intrinsic plastic behavior of metallic glasses: the roles of atomic configuration and alloy composition. Acta Mater.

[54] Zhong L, Wang J, Sheng H, Zhang Z, Mao SX (2014). Formation of monatomic metallic glasses through ultrafast liquid quenching. Nature.

[55] Li J, Dai X, Liang S, Tai K, Kong Y, Liu B (2008). Interatomic potentials of the binary transition metal systems and some applications in materials physics. Phys Rep.

[56] Li J, Dai Y, Cui Y, Liu B (2011). Atomistic theory for predicting the binary metallic glass formation. Mater Sci Eng R Rep.

[57] Şopu D, Foroughi A, Stoica M, Eckert J (2016). Brittle-to-ductile transition in metallic glass nanowires. Nano Lett.

[58] Şopu D, Soyarslan C, Sarac B, Bargmann S, Stoica M, Eckert J (2016). Structure-property relationships in nanoporous metallic glasses. Acta Mater.

[59] Li QK, Li M (2007). Assessing the critical sizes for shear band formation in metallic glasses from molecular dynamics simulation. Appl Phys Lett.

[60] Shimizu F, Ogata S, Li J (2006). Yield point of metallic glass. Acta Mater.

[61] Ogata S, Shimizu F, Li J, Wakeda M, Shibutani Y (2006). Atomistic simulation of shear localization in Cu–Zr bulk metallic glass. Intermetallics.

[62] Li M, Wang CZ, Hao SG, Kramer MJ, Ho KM (2009). Structural heterogeneity and medium-range order in zr$$_{x}$$ cu$$_{100-x}$$ metallic glasses. Phys Rev B.

[63] Wu ZW, Li MZ, Wang WH, Liu KX (2015). Hidden topological order and its correlation with glass-forming ability in metallic glasses. Nat Commun.

[64] Peng HL, Li MZ, Wang WH, Wang CZ, Ho KM (2010). Effect of local structures and atomic packing on glass forming ability in cu$$_x$$zr$$_{100-x}$$ metallic glasses. Appl Phys Lett.

[65] Glänzel W, Schubert A (2005). Analysing scientific networks through co-authorship.

[66] Didegah F, Thelwall M (2013). Which factors help authors produce the highest impact research? Collaboration, journal and document properties. J Informet.

[67] Wu Y, Wang B, Hu Y, Lu Z, Li Y, Shang B, Wang W, Bai H, Guan P (2017). The critical strain—a crossover from stochastic activation to percolation of flow units during stress relaxation in metallic glass. Scripta Mater.

[68] Lü Y, Guo C, Huang H, Gao J, Qin H, Wang W (2021). Quantized aging mode in metallic glass-forming liquids. Acta Mater.

[69] Wu WP, Şopu D, Yuan X, Adjaoud O, Song K, Eckert J (2021). Atomistic understanding of creep and relaxation mechanisms of cu64zr36 metallic glass at different temperatures and stress levels. J Non-Cryst Solids.

[70] Zhang H, Sun H, Pan S, Şopu D, Peng C, Zhao K, Song K, Yuan S, Qiao J, Wang L, Eckert J (2021). Origin of structural heterogeneity in Zr–Co–Al metallic glasses from the point of view of liquid structures. J Non-Cryst Solids.

[71] Ruestes CJ, Alhafez IA, Urbassek HM (2017). Atomistic studies of nanoindentation-a review of recent advances. Curr Comput-Aided Drug Des.

[72] Simdyankin SI, Dzugutov M, Taraskin SN, Elliott SR (2001). Connection between vibrational dynamics and topological order in simple glasses. Phys Rev B.

[73] Falk ML, Langer JS (2000). From simulation to theory in the physics of deformation and fracture. MRS Bull.

[74] Varnik F, Bocquet L, Barrat JL (2004). A study of the static yield stress in a binary Lennard–Jones glass. J Chem Phys.

[75] Lund AC, Schuh CA (2003). Yield surface of a simulated metallic glass. Acta Mater.

[76] Arman B, Luo SN, Germann TC, Çağın T (2010). Dynamic response of cu$$_{46}$$zr$$_{54}$$ metallic glass to high-strain-rate shock loading: Plasticity, spall, and atomic-level structures. Phys Rev B.

[77] Wen P, Demaske B, Spearot DE, Phillpot SR (2018). Shock compression of cu$$_{x}$$zr$$_{100-x}$$ metallic glasses from molecular dynamics simulations. J Mater Sci.

[78] Albe K, Ritter Y, Şopu D (2013). Enhancing the plasticity of metallic glasses: Shear band formation, nanocomposites and nanoglasses investigated by molecular dynamics simulations. Mech Mater.

[79] Amigo N, Sepulveda-Macias M, Gutierrez G (2019). Enhancement of mechanical properties of metallic glass nanolaminates via martensitic transformation: atomistic deformation mechanism. Mater Chem Phys.

[80] Scudino S, Bian JJ, Shakur Shahabi H, Şopu D, Sort J, Eckert J, Liu G (2018). Ductile bulk metallic glass by controlling structural heterogeneities. Sci Rep.

[81] Shimono M, Onodera H (2005). Structural relaxation in supercooled liquids. Mater Trans.

[82] Peng HL, Li MZ, Wang WH, Wang C, Ho KM (2010). Effect of local structures and atomic packing on glass forming ability in cuxzr100-x metallic glasses. Appl Phys Lett.

[83] Lad KN, Jakse N, Pasturel A (2012). Signatures of fragile-to-strong transition in a binary metallic glass-forming liquid. J Chem Phys.

[84] Shi Y, Falk M (2006). Does metallic glass have a backbone? the role of percolating short range order in strength and failure. Scr Mater.

[85] Cameron KK, Dauskardt RH (2006). Fatigue damage in bulk metallic glass I: simulation. Scr Mater.

[86] Pan SP, Qin JY, Wang WM, Gu TK (2011). Origin of splitting of the second peak in the pair-distribution function for metallic glasses. Phys Rev B.

[87] Wang C, Wong C (2012). Interpenetrating networks in Zr–Cu-al and Zr–Cu metallic glasses. Intermetallics.

[88] Priezjev NV (2021). Accelerated rejuvenation in metallic glasses subjected to elastostatic compression along alternating directions. J Non-Cryst Solids.

[89] Zhu B, Huang M, Li Z, Du J, Sun Y, He M, Zhang Y (2021). Enhanced ductility in cu64zr36 metallic glasses induced by prolonged low-energy ion irradiation: a molecular dynamics study. J Alloys Compd.

[90] Shang B, Wang W, Greer AL, Guan P (2021). Atomistic modelling of thermal-cycling rejuvenation in metallic glasses. Acta Mater.

[91] Amigo N (2022). Role of high pressure treatments on the atomic structure of cuzr metallic glasses. J Non-Cryst Solids.

[92] Hua D, Ye W, Jia Q, Zhou Q, Xia Q, Shi J, Deng Y, Wang H (2020). Molecular dynamics simulation of nanoindentation on amorphous/amorphous nanolaminates. Appl Surf Sci.

[93] Doan DQ, Fang TH, Chen TH (2020). Nanotribological characteristics and strain hardening of amorphous cu64zr36/ crystalline cu nanolaminates. Tribol Int.

[94] Avila KE, Küchemann S, Alabd Alhafez I, Urbassek HM (2020). An atomistic study of shear-band formation during cutting of metallic glasses. J Appl Phys.

[95] Vardanyan VH, Avila KE, Küchemann S, Urbassek HM (2021). Interaction of dislocations and shear bands in cutting of an amorphous-crystalline bilayer: an atomistic study. Comput Mater Sci.

[96] Yuan S, Song X, Branicio PS (2020). Tuning the mechanical properties of shape memory metallic glass composites with brick and mortar designs. Scripta Materialia.

[97] Yuan S, Branicio PS (2020). Gradient microstructure induced shear band constraint, delocalization, and delayed failure in cuzr nanoglasses. Int J Plast.

[98] Du H, Li N, Brown MA, Peng Y, Shuai Y (2014). A bibliographic analysis of recent solar energy literatures: the expansion and evolution of a research field. Renew Energy.

[99] Sachini E, Sioumalas-Christodoulou K, Chrysomallidis C, Siganos G, Bouras N, Karampekios N (2021). Covid-19 enabled co-authoring networks: a country-case analysis. Scientometrics.

[100] Dumaz M, Boucher R, Marques MAL, Romero AH (2021). Authorship and citation cultural nature in density functional theory from solid state computational packages. Scientometrics.

[101] BdPFe Fonseca, Sampaio RB, Fonseca MVdA, Zicker F (2016). Co-authorship network analysis in health research: method and potential use. Health Res Policy Syst.

[102] Liu H, Song J, Wang G (2021). A scientometric review of smart construction site in construction engineering and management: analysis and visualization. Sustainability.

